# Rapid Review to Inform the Rehabilitation and Reintegration of Child Returnees from the Islamic State

**DOI:** 10.5334/aogh.2835

**Published:** 2020-06-19

**Authors:** Stevan Weine, Zachary Brahmbatt, Emma Cardeli, Heidi Ellis

**Affiliations:** 1 University of Illinois at Chicago, US; 2 Boston Children’s Hospital, US

## Abstract

**Background:**

An estimated 49,000 women and children who lived in the Islamic State are being held in the Al-Hol refugee camp in Syria. Several countries have repatriated some of these women and children, though most have thus far refused to do so. Many countries are asking whether it is possible to successfully rehabilitate and reintegrate this group and how the evidence base could inform their approach.

**Objective:**

The overall objective of this paper is to inform the rehabilitation and reintegration of child returnees from the Islamic State by rapidly reviewing the evidence on children exposed to trauma and adversity.

**Methods:**

A rapid review was conducted to identify pertinent evidence regarding outcomes, risk and protective factors, and interventions and to build a framework that could guide policies and practices. Prior work in the areas of refugee children, war-impacted children, child criminal gang members, child victims of maltreatment, and child victims of sex trafficking was reviewed. Evidence was collected and analyzed from 31 prior reviews and studies.

**Findings:**

The Rehabilitation and Reintegration Intervention Framework (RRIF) incorporates five levels (individual, family, educational, community, and societal) and identifies five primary goals: 1) promoting individual mental health and well-being; 2) promoting family support; 3) promoting educational success; 4) promoting community support; and 5) improving structural conditions and protecting public safety. Implementing this framework requires public-private partnership with extensive civil society involvement.

**Conclusions:**

Rehabilitation and reintegration programs should be based on the evidence of prior work with children exposed to trauma and adversity. RRIF defines a multi-level approach that encompasses promoting individual mental health and well-being, family support, educational success, community support, structural conditions, and public safety. Further multi-disciplinary research is needed to develop evidence in several identified areas concerning child health and developmental problems, family custody, faith and religiosity, and violent extremism assessment and prevention.

## Introduction

The Islamic State (IS) terrorist organization orchestrated a calculated recruitment strategy that drew nearly 30,000 men and women from countries all over the world [1]. Many of these men and women brought along their children, while even more gave birth to children during their time in the IS [2,3]. After a strong counter-offensive that depleted the majority of IS forces, many of the women and children were left behind, confined to refugee camps and detainment centers [2].

In the Syrian Al-Hol refugee camp, there are nearly 49,000 children who have been refused by their home communities and confined to abysmal conditions [4]. These include about 1,300 children from European countries [5]. Many countries are afraid that the children will pose a security risk if they are allowed to return. The December 2019 stabbing in London by a convicted Islamist terrorist, released after serving six years in prison, underlined those fears [6]. However, inaction may breed an even larger humanitarian and security crisis.

Thus far, a relatively small number of children and mothers have been repatriated to their countries. The United States has accepted only about a dozen returnees [7]. Kazakhstan, a Central Asian country and former Soviet republic, repatriated more than 447 children and 161 mothers, along with 30 adult male fighters, according to Kazakhstan and U.S. officials. The government developed a national rehabilitation and reintegration program with local nongovernmental organizations to support this effort. In Kazakhstan, bringing children and their mothers back is framed as a humanitarian and moral issue. These children are regarded as victims, even if some were taught extremist ideology or how to use weapons. Even women who followed their husbands or fathers to the IS were not necessarily committed terrorists. The Kazakhstanis want to return these children and mothers to the motherland and move them away from violent extremism. This involves much more than just the legal act of repatriation. Most children and women are being supported outside of the criminal justice system and are being provided with community-based psychosocial support, specialized schooling, job training, and family assistance.

## Background

There is a shortage of international good practices and frameworks to guide rehabilitation and reintegration of child returnees [8]. As a result, many countries are currently in the process of determining whether or not to repatriate women and children; however, they are making these decisions with limited empirical guidance or practice-based evidence on which to draw. Receiving countries should develop and implement rehabilitation and reintegration programs that are evidence- or best practice-based, drawing on lessons learned from multidisciplinary programs that have been implemented to help other children, then adapting these practices to the local context.

Existing literature in the clinical, community, and social sciences has examined children and their families who have been impacted by a number of different traumatic and adverse situations. These include: refugee children, war-impacted children, child criminal gang members, child victims of maltreatment, and child victims of sex trafficking. None of these other groups are exactly the same as the children who are returning from the IS, but nonetheless there are important similarities, as described below.

In reviewing the literature, we first identified how the research articles typically define the following key components: *outcomes* (the individual or social changes expected as a result of the practice or program); *risk factors* (characteristics at the biological, psychological, family, community, or cultural level that precede and are associated with a higher likelihood of negative outcomes); *protective factors* (multi-level characteristics that reduce a risk factor’s adverse impact); and *evidence-based practices* (interventions that have been researched academically or scientifically, been proven effective, and replicated by more than one investigation or study).

The overall objective of this paper is to inform the rehabilitation and reintegration of child returnees from the Islamic State by rapidly reviewing the evidence on children exposed to trauma and adversity.

## Method

We conducted a rapid review of the literature on refugee children, war-impacted children, child soldiers, child criminal gang members, child victims of maltreatment, and child victims of sex trafficking. These six areas were chosen because each area had adequate scientific literature and because children’s exposure to trauma and adversity in each area overlapped significantly with that of child returnees from the IS, as represented in Table 1.

**Table 1:** Overlap of child returnees with other types of childhood trauma and adversity.

	Refugee Children	War- Impacted Children	Child Criminal Gang Members	Child Victims of Maltreatment	Child Victims of Sex Trafficking

**Prior Childhood Adversity & Trauma**	X	X	X	X	X
**Family Violence**	X	X	X	X	X
**Community/Political Violence**	X	X	X		X
**Combat Involvement**		X	X		
**Victim of Indoctrination**		X	X		X
**Family Loss & Separation**	X	X	X	X	X
**Displacement & Adjustment Stressors**	X	X			X

Rapid reviews are a way of gathering evidence to inform policy and program decision making by streamlining the methods of a systematic literature review [9]. Compared with a systematic review, a rapid review includes fewer numbers of articles and has a preference for including existing systematic reviews.

In order to assess the relevant literature, PubMed and EBSCO were searched for English-language articles using the following keywords in various combinations: refugees, war-impacted, child soldiers, terrorism, criminal gangs, maltreatment, sex trafficking, risk factors, protective factors, and research. The reference sections of these articles were also examined to identify additional relevant articles. Given the first author’s extensive prior work on this topic, files from past searches were examined and relevant articles included. The authors acknowledge that despite extensive literature searches, some relevant articles may have been omitted. A total of 73 articles or chapters were reviewed for possible inclusion, of which a total of 31 were chosen, including 14 reviews.

From a preliminary review of the entire database of articles, we first identified three key questions that could apply to each of the above groups: 1) How are positive outcomes defined? 2) What are the multi-level (individual, family, community, society) risk and protective factors associated with positive outcomes? 3) What evidence-based, best, or emerging practices are being used? Further, a comprehensive table and summary narratives were developed to answer each of these questions in each of the five areas of literature reviewed.

In order to build the framework, lists of potentially modifiable outcomes, risk and protective factors, and practices were grouped into the following five levels of goals: 1) promoting individual mental health and well-being; 2) promoting family support; 3) promoting educational success; 4) promoting community support; and 5) improving structural conditions and protecting public safety. The findings were further refined into an overall framework called the Rehabilitation and Reintegration Intervention Framework (RRIF), which identified the risk and protective factors at each of the five levels, associated levers of community resilience, and policy and program priorities.

## Findings

The results of the rapid review are summarized in Table 2 and described in the paragraphs below.

**Table 2:** Rapid review results.

Population	Studies	Outcomes (Goal)	Risk Factors	Protective Factors	Intervention Strategies

**Refugee Children**	Review (Fazel et al. 2011)	Decreased psychological disturbance and adverse mental health symptoms	Exposure to violence Poor parental health Discrimination	Family cohesion Parental health Social support Community acceptance School safety and belonging	Psychotherapy combined with structural interventions (housing and skills training) Equitable access.
	Review (Ehntholt & Yule 2006)	Decreased adverse psychiatric symptoms	Traumatic events Post-migration stresses Poor parental health	Family cohesion and adaptability Social support Belief systems	Phased model approach – establish safety and trust, trauma therapy, then reintegration Cognitive behavior treatment (CBT) Narrative exposure therapy (NET) Testimony psychotherapy
	Review N. Korean Refugees (Lee et al. 2017)	Decreased adverse psychiatric symptoms Psychological adaptation	Strenuous immigration process Acculturative stress	Social support	—
	7 Countries (Mohamed & Thomas 2017)	“Ability to bounce back from adversity and even thrive in the face of challenges”	Bullying and racism Poor connectedness to the community Language barriers	Social support and friendships Education Teacher support	Education and care plans for training NET Robust anti-bullying policies CBT Partnerships with parents Cultural acceptance and celebration programs
	Arab Refugees (Kira et al. 2013)	Good health outcomes in spite of adversity	Stigmatization Exposure to trauma Acculturation stressors	Intact family Religion and religious leaders Perception of self-control Forgiveness Perception of retributive justice	Family therapy CBT NET Psycho-educational group therapy Assertiveness training Trauma systems therapy Recreational activities Multisystemic therapy Structural ecosystems theory Rights-based care
	Canadian and Southeast Asian Refugees (Rousseau et al. 1998)	Increased prosocial behaviors Decrease in internalizing symptoms	Exposure to trauma Parental depression Family conflict Family separation Family trauma	Social support Network of peers Sponsorship	—
	Review (Eruyar et al. 2018)	Increase in resilient behavior and absence of psychopathy	Poor parental health Discrimination School Exclusion Criminality Absence of environmental safety	Parental support Family connectedness Social support	Parent-child therapy and family-based intervention CBT-focused teaching recovery techniques program Interpersonal group psychotherapy NET Creative therapy Eye movement desensitization and reprocessing therapy Multimodal interventions
**War-impacted Children**	Afghanistan (Panter-Brick & Eggerman 2011)	Positive social adjustment and functional behavior in the midst of conflict	Domestic violence Community and political violence Family health Economic hardship Separation from close friend Social suffering Overcrowding	School attendance Family unity Strong family values Faith Social support networks Better living conditions	Child and family-focused mental health interventions
	Palestine (Nguyen-Gillham et al. 2008)	Positive health outcomes in spite of dehumanizing conditions	Chronic exposure to violence Economic hardship Lack of environmental security/comfort Humiliation	Networks of social support (friends and family) School attendance Political activism/identity	Fostering new social networks
	Review (Karadzhov 2015)	Absence of psychopathy Increased prosocial behaviors	Economic hardship Stigmatization Domestic violence Motivation to seek revenge Acculturation Inequitable access to facilities	School attendance Community acceptance High SES Perceived spiritual support Social intelligence Empathy and hope Cultural affiliation Social Support Political Participation	Community resilience and rehabilitation Trauma counseling
	Review (Tol et al. 2013)	Good mental health and developmental outcomes	—	Optimism Mental flexibility and social intelligence Religiosity Parental monitoring and support Safe home environment School retention Peer social support Community Acceptance	Develop supportive socio-ecological context Don’t over idealize cultural resources
	Review (Jordans et al. 2009)	Reduction in symptoms	Family separation Community tension	Parental support and interaction Strong family roles Social support Recreational activities Secure school environment Community awareness Group cohesion	Creative-expressive, recreational, psycho-education activities Narrative exposure therapy Trauma group psychotherapy Dance and movement therapy CBT Group interpersonal therapy Parent-child interaction therapy Teacher and health worker sensitization
	Review (Williams 2007)	Adapt psychologically, emotionally, and physically well in spite of adversity	Exposure to trauma Family Loss Poor parental practices Poor family health Loss of places of education and social gathering Loss of routine	Intelligence and temperament Family relationships and support Social and institutional support	Culturally sensitive approach Psychological first aid Community mental health services Specialist psychiatric and psychotherapeutic services Engage in recreational activities
	Sierra Leone (Betancourt 2010)	Increases in prosocial behaviors	War trauma Stigmatization Daily hardship	Community acceptance Social support School attendance	Community sensitization and acceptance campaigns
	Colombia (Cortes & Buchanan 2007)	Exhibition of mild or no trauma related symptoms	—	Autonomy Self-confidence Interpersonal awareness Empathy Sense of hope Spirituality Morality	—
**Child Criminal Gang Members**	Ottawa (Hastings et al. 2011)	Successful disengagement from gang and prosocial behavior	Fear of retaliation Low neighborhood or school attachment Family disorganization Social disorganization Commitment to delinquent peers Fear of retaliation Lack of education or employment Stigmatization	Access to education Healthy family relationships Safe Environment	Training and employment programs Combination of prevention, intervention, and suppression Peer mentoring
	U.K. (Harris et al. 2011)	Desistance from gang activities	Attachment to gang Localization Stigmatization	Maturation Family Access to employment Social relationships	Resettlement Psychosocial treatment
	Arizona (Pyrooz & Decker 2011)	Desistance from gang activities	Embeddedness	Family responsibilities Job responsibilities Resettlement Maturation	Community and CJ supported desistance
	Review (Carson & Vecchio 2015)	Desistance from gang activities	Marital discord Police harassment Fear of rival gangs Unemployment	Maturation Disillusionment Official sanctions Police contact Spirituality and religiosity Encouragement from teachers, parents or adults Meaningful employment Romantic relationships Family responsibilities Resettlement	—
	Review (O’Brien et al. 2013)	Desistance from youth gang activities	—	Increased parental monitoring Social skills Commitment to school Attachment to mentors Family cohesiveness Maturation Traumatic Events	Phoenix gang intervention program CBT Motivational interviewing
	Glasgow, Scotland (Gormally 2014)	Desistance from criminal youth activities	Investment in the gang	De-identification Maturation Employment Religion	—
**Child Victims of Maltreatment**	Review (Afifi & MacMillan 2011)	Absence of psychopathy, social functioning, positive self-esteem	Parental rejection Self-blame	Less unilateral parent decision making Stable family Normal adolescent relationships Good adult friendships Greater commitment to school Family cohesion Intelligence Life satisfaction Self-efficacy Optimism	Trauma informed clinical care
	Review (Marriott et al. 2014)	Few long-term negative outcomes	Early abuse	Stable family environment Positive parenting practices Strong friendships Adulthood relationship Positive school experiences Religious participation	Focus on inner resources (internal resilience from strong family, friends, adult network) Health promotion initiatives and social programs
	United States (Folger & Wright 2013)	Reduction in symptoms of depression, anxiety and hostility	Dating abuse Cumulative maltreatment	Perceived support from family and friends Support from a partner	—
	Review (Domhardt et al. 2015)	Normal functioning and positive adaptation	—	Externalizing blame Education and school engagement Emotional intelligence Emotional attachment to family member Religiosity Leisure activities High SES Stable family Positive parenting Community social support School safety	Trauma focused cognitive behavior therapy Educational engagement Facilitate interpersonal trust Enhance social support provided by family members
	United States (Greenfield & Marks 2010)	Long-term resilience and positive health outcomes	Parental violence Psychological violence	Sense of community	—
	Women Survivors (Hyman & Williams 2001)	Absence of psychological difficulties	Personal substance abuse Parental substance abuse Criminal activity Re-victimization	Self-esteem Intimate relationships Community participation Adherence to community standards Stable family Family Support Social Support Education	—
**Child Victims of Sex Trafficking**	Review (Muraya & Fry 2015)	Restoration of the physical and mental health of victims	Drug use Social detachment Social isolation Connections to traffickers Discrimination (in terms of receiving services)	Safe environment Education Social support Employment (job training) Adequate housing	Trauma informed services STOP-IT Chicago program rights -based care Individual counseling Group sessions Creative therapies Psychiatric care Trauma-focused CBT Appropriate medical care Holistic aftercare services
	Dissertation (Evans 2019)	Recovery from trauma and improved health outcomes	Shame Dissociation Poverty Absence of social support network Stigmatization Drug use Forced involvement in CJ process Attachment to traffickers Unhealthy family relationships	Community support Adequate housing Strong family relationship Spirituality Structure and safety Personal growth Financial stability Education	Culturally appropriate services Language services Mental health care Housing Job training Trauma-focused CBT Public awareness campaign Legislation
	Review (Abu-Ali & Al-Bahar 2011)	Successful reintegration Absence of trauma-related symptoms	Early separation from caregivers Attachment to trafficker Marginalization Family punishment	Strong identity Cultural identity	Integrated psychotherapy and social justice model

### Refugee Children

Positive outcomes for refugee children were defined as an absence of psychological difficulties and adverse mental health outcomes.

The studies identified eleven risk factors for refugee children at multiple levels (as indicated in parentheses): exposure to violence, chronic illness, behavioral issues, developmental disorders, strenuous immigration policies, housing issues, and acculturative stress such as language barriers (individual level); family conflict and trauma, family separation, and poor parental health (family level); stigmatization and discrimination, including bullying and racism, and high crime rates (community level) [10,11,12,13,14,15,16]. For example, Rousseau and Drapeau demonstrated among child refugees from Southeast Asia that poor parental health and depression were risk factors for internalizing symptoms [15].

We identified ten protective factors for refugee children that could similarly be organized by socio-ecological levels: positive self-esteem, social flexibility, forgiveness, perception of self-control, perception of retributive justice and spirituality/religiosity (individual level); family cohesion and adaptability, strong parental health, and higher household socioeconomic status (family level); strong social support networks, community acceptance, education, presence of religious leaders, and safe environments (community level) [10,11,12,13,14,15,16]. For example, Kira et al. found among Arab refugees that family cohesion and the preservation of family bonds protected against trauma-related symptoms [14].

Helpful clinical, educational, and community-based interventions were identified as follows, with attention to the socio-ecological levels at which an intervention is delivered: cognitive behavior therapy (CBT), narrative exposure therapy (NET), testimony psychotherapy, psycho-educational group therapy, eye movement desensitization and reprocessing therapy, creative therapy (individual level); family therapy, parent-child focused therapy (family level); school sensitization, robust anti-bullying policies (school level); cultural and spiritual celebrations and programs, and community sensitization (community level); and housing, training, and employment programs (governmental/non-governmental levels) [10,12,13,15,16]. In particular, multimodal approaches that address the mental health needs of refugee children in the context of the social environment are increasingly seen as holding promise [16], one example being Trauma Systems Therapy for Refugees [17].

### War-Impacted Children

War-impacted children included child soldiers and other children impacted by warfare and extremism in their countries. For example, one study focused on children in Afghanistan exposed to extremism and prolonged conflict [18] and another on former child soldiers in Sierra Leone [19].

Positive outcomes for war-impacted children were an absence of psychopathy, symptom reduction, and increased prosocial behaviors.

We identified thirteen risk factors for war-impacted children at multiple levels: chronic exposure to violence, vengefulness, presence of a physical or mental disability (individual level); economic hardship, maltreatment, family separation (family level); stigmatization and humiliation, lack of work for mothers and other adult family members, acculturation stressors, overcrowding, inequitable access to facilities, and the lack of environmental safety (community level) [18,19,20,21,22,23]. For instance, community and family violence led to poor mental health in Afghani war-impacted children [18].

We identified sixteen protective factors for war-impacted children at multiple levels: sense of humor, empathy, positive self-esteem, social intelligence, temperament, and optimism (individual level); a safe home environment, strong family roles and values, unity, and family acceptance and support (family level); social support, education, community acceptance/awareness, institutional support, political participation, and religious and cultural affiliations (community level) [18,19,20,21,22,23,24,25]. In a longitudinal study of former child soldiers in Sierra Leone, Betancourt et al. found that community acceptance and retention in school led to an increase in prosocial behaviors and lower levels of internalizing problems [19]. Political activism contributed to resilience and recovery for war-impacted children in Palestine [20].

Helpful intervention strategies included CBT, NET, trauma group psychotherapy, dance and movement therapy (individual level); parent-child interaction therapy (family level); teacher sensitization and trauma-informed education (school); community resilience and sensitization, anti-discrimination campaigns, medical care and health worker sensitization (community level) [18,19,20,21,22,23,24]. For example, Ertl et al. found that community-based narrative exposure therapy was effective for PTSD for former child soldiers in Uganda [26].

### Child Criminal Gang Members

While this rapid review is concerned with children, the literature on criminal gang members includes both children and adults. Positive outcomes for former gang members were desistance from gang-related activities and increased prosocial behavior. For example, Gormally analyzed the factors that promoted desistance from youth gang behaviors in Glasgow, such as maturation [27].

We identified six risk factors for former criminal gang members at multiple levels: commitment to delinquent peers, fear of gang retaliation, lack of education and employment (individual level); family disorganization and discord including child maltreatment (family level); stigmatization, lack of access to quality education and employment (community level) [27,28,29,30,31].

We identified nine protective factors for former criminal gang members at multiple levels: maturation, disillusionment (individual level); healthy family relationships and responsibilities (family level); access to education, meaningful employment, resettlement, spirituality/religiosity, strong network of support and encouragement (community level) [27,28,29,30,31,32]. For example, O’Brien et al., in their systematic review, observed that maturation amongst youth gang members encouraged gang desistance [32].

Helpful intervention strategies included psychosocial treatment, peer mentoring, community and criminal justice-supported desistance, resettlement, education, training, and employment programs [28,29,31,32]. Hastings et al. found that psychosocial programming that included components of CBT was necessary to diminish mental health symptoms following gang desistance for Ottawan youth [28].

### Child Victims of Maltreatment

Positive outcomes for child victims of maltreatment were an absence of psychopathy, increased social functioning, and fewer long-term negative outcomes.

We identified eight risk factors for child victims of maltreatment at multiple levels: substance abuse and earlier instances of maltreatment (individual level); parental rejection and economic hardship (family level); stigmatization, discrimination, lack of access to care, community violence, poor education, and social isolation (community level) [33,34,35,36,37]. For example, Folger & Wright found that child victims of maltreatment who lacked support from family and friends reported more symptoms of anxiety and depression than those who had social supports [35].

We identified seventeen protective factors for child victims of maltreatment at multiple levels: externalizing blame, emotional intelligence, and life satisfaction (individual level); consistent parental employment, less unilateral parent decision-making, family cohesion, positive parenting practices, emotional attachment to a family member, and high SES (family level); access to care and social services, perceived social support, strong friendships, mentors outside the family, school attendance and safety, positive school experiences, adequate housing, spirituality/religiosity, and participation in leisure activities (community level) [33,34,35,36,37,38]. For example, Hyman and Williams discovered that the perception of good parental practices and a strong peer network was protective against poor mental health outcomes for women who were child victims of abuse [37]. Greenfield and Marks found that a sense of community promoted resilience in child victims of maltreatment [36].

Helpful intervention strategies included trauma-focused CBT, educational engagement, health promotion initiatives, and resilience programming that espouses a broad network of support to facilitate interpersonal trust [33,34,38]. For example, Domhardt et al. found that trauma-focused CBT was effective for child victims of sexual abuse [38].

### Child Victims of Sex Trafficking

Positive outcomes for sex trafficking victims were a recovery from trauma and improved health outcomes.

We identified eleven risk factors for sex trafficking victims at multiple levels: substance abuse, shame, dissociation and detachment, attachment to traffickers, and early separation from caregivers (individual level); conflictual family relationships and family punishments (family level); stigmatization, discrimination, social isolation, forced involvement in the criminal justice process, and poverty (community level) [39,40,41]. For example, Evans found that forcing victims of sex trafficking to participate in the criminal justice process against their traffickers could cause re-traumatization [40].

We identified ten protective factors for sex trafficking victims at multiple levels: personal growth and strong identity (individual level); strong family relationships (family level); community support, structure, and safety, employment and financial stability, adequate housing, education, spirituality/religiosity, and cultural identity (community level) [39,40,41]. Abu-Ali & Al-Bahar found that promoting cultural identity for former sex trafficking victims promoted empowerment and protected against victimization [41].

Helpful intervention strategies include trauma-focused CBT, individual counseling, group trauma therapy, rights-based care, creative therapies, language, and culturally appropriate services, and medical care (individual level); housing, job training, employment, public awareness campaigns, and legislation (community level) [38,39,40,41]. For example, Fry & Muraya [39] found that rights-based care was central to the treatment and recovery of child victims of sex trafficking.

### Rehabilitation and Reintegration Intervention Framework

**Figure 1:**
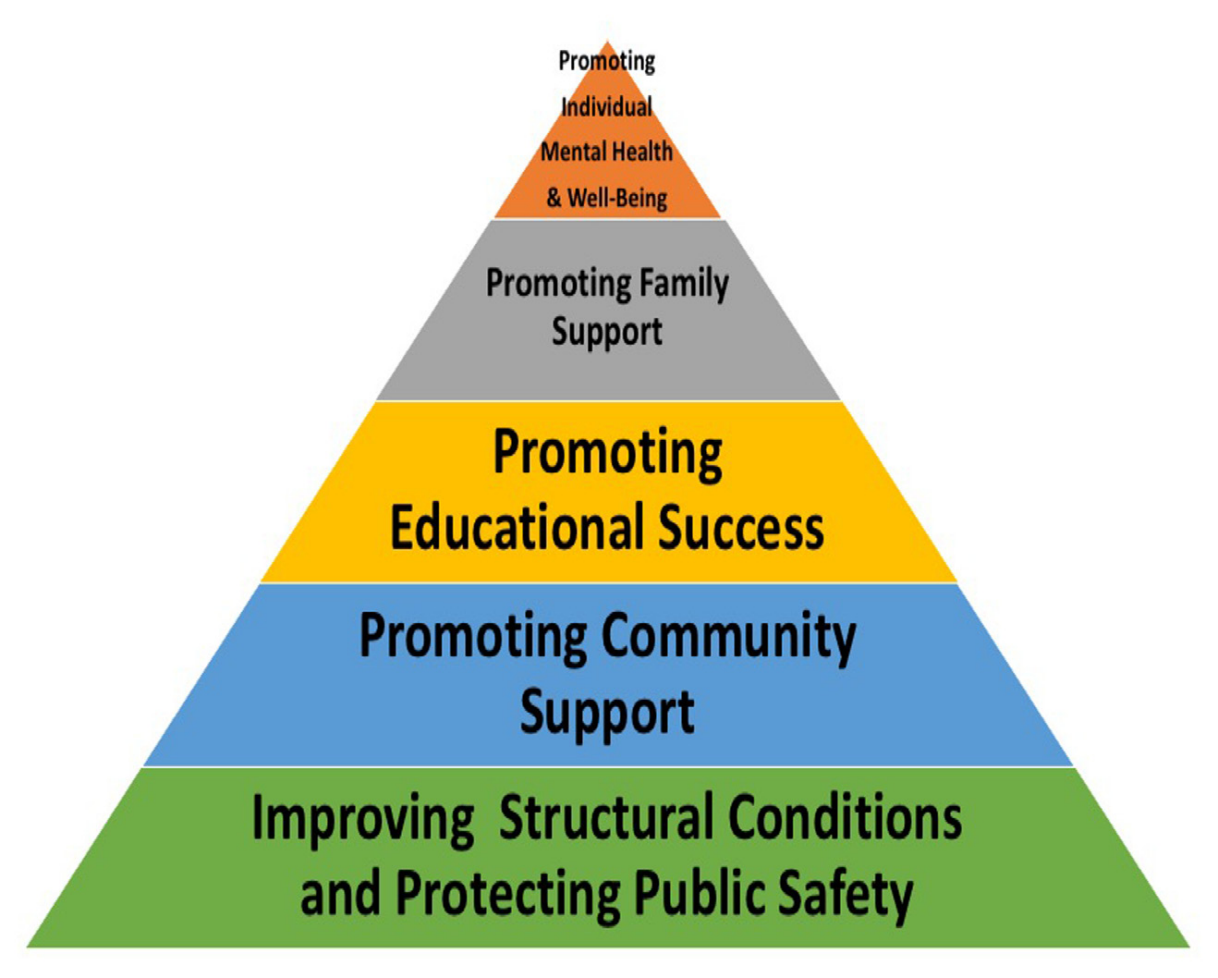
The rehabilitation and reintegration intervention framework (RRIF).

Based upon the evidence from the 31 prior reviews and studies, and also informed by our field experience with in several countries rehabilitation and reintegration programs, we built the Rehabilitation and Reintegration Intervention Framework (RRIF). It consists of five distinct levels (Figure 1 below). The RRIF emphasizes a multilevel approach, implying that activities are needed at each level in order to succeed with the rehabilitation and reintegration of child returnees.

The framework identifies five primary goals for rehabilitation and reintegration that encompass multiple levels: individual, family, school, community, governmental, and non-governmental organizations. The primary goals are: 1) promoting individual mental health and well-being; 2) promoting family support; 3) promoting educational success; 4) promoting community support; and 5) improving structural conditions and protecting public safety. Note: the proportionality of this figure is not intended to convey that the larger, lower levels of the model are more important.

**Figure 2:**
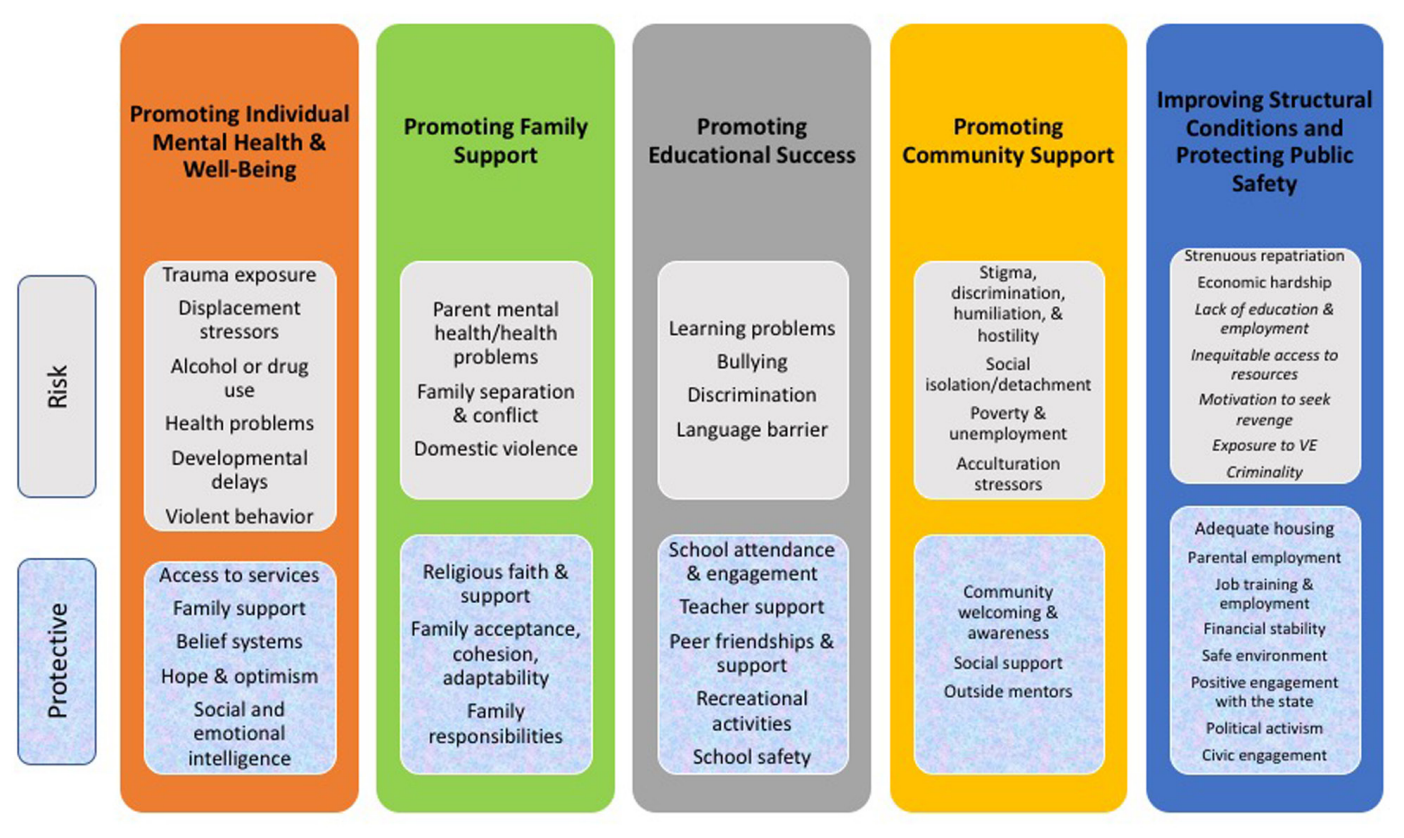
Risk and protective factors for the rehabilitation and reintegration of child returnees.

Then, we sorted the risk and protective factors identified through the review into those five levels (Figure 2 below). RRIF illustrates which risk and protective factors will likely impact outcomes at each level.

**Figure 3:**
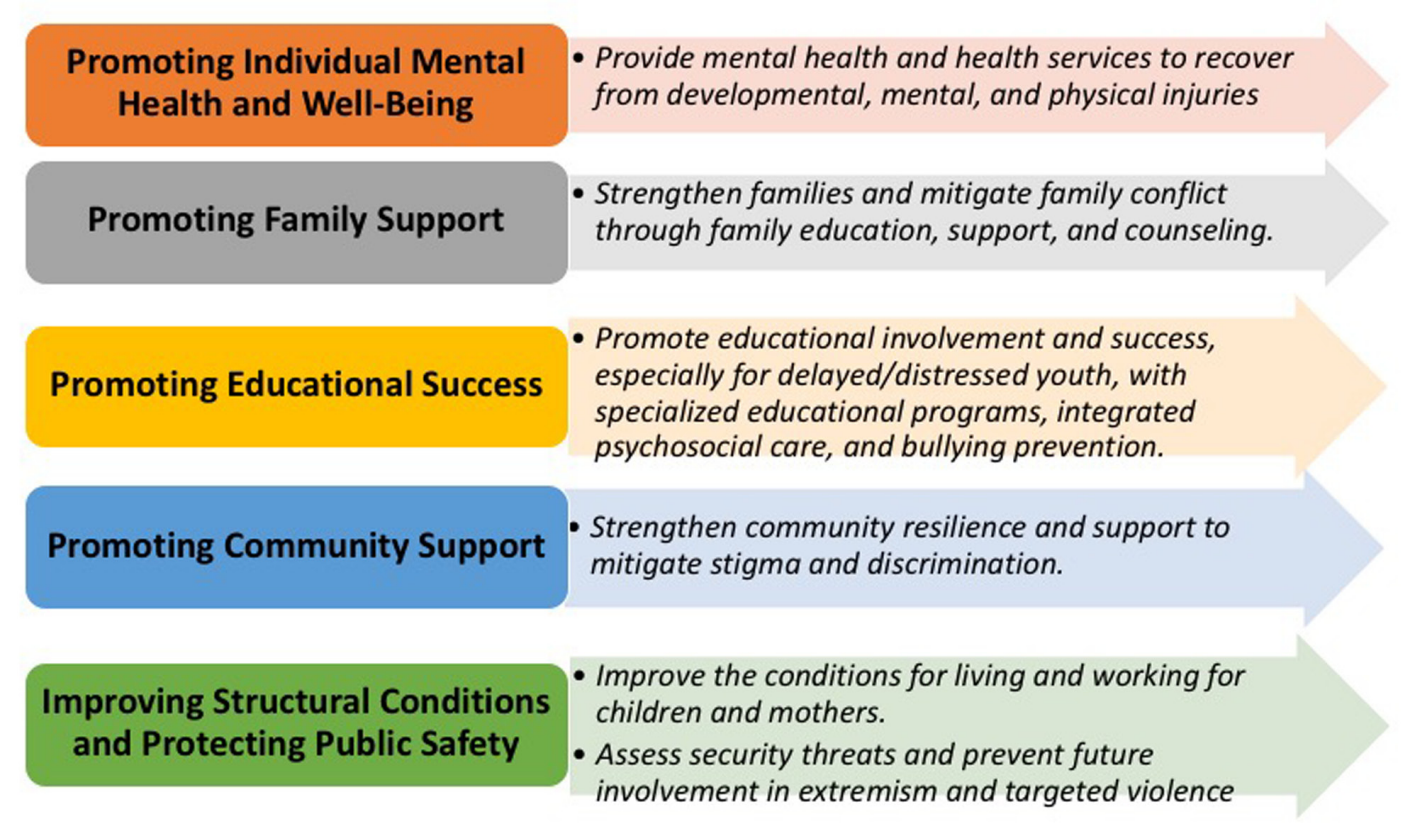
Policy goals for the rehabilitation and reintegration of child returnees.

Then, we drew upon the evidence-based practices from the review to determine the overall policy goals for each level. These goals optimize risk and protective factors so as to achieve the best outcomes for child returnees (Figure 3 below). Improving structural conditions and protecting public safety can be achieved through improving the conditions for growing, living, working, and aging for the child and mother and assessing for and preventing involvement in violent extremism and targeted violence. Promoting community support can be achieved through strengthening community resilience and mitigating against stigma and discrimination. Promoting educational success can be achieved through advocating for special education services that directly target identified learning problems or gaps in education, promoting individual and parental school involvement, especially for youth with developmental delays or significant social-emotional problems, and protecting against bullying and other forms of discrimination. Promoting family support can be achieved through strengthening family bonds and mitigating family conflict through family education, support, and counseling. Promoting individual mental health and well-being can be achieved through providing trauma-informed mental health and health services to help individuals recover from health, mental health, and developmental or physical injuries.

**Figure 4:**
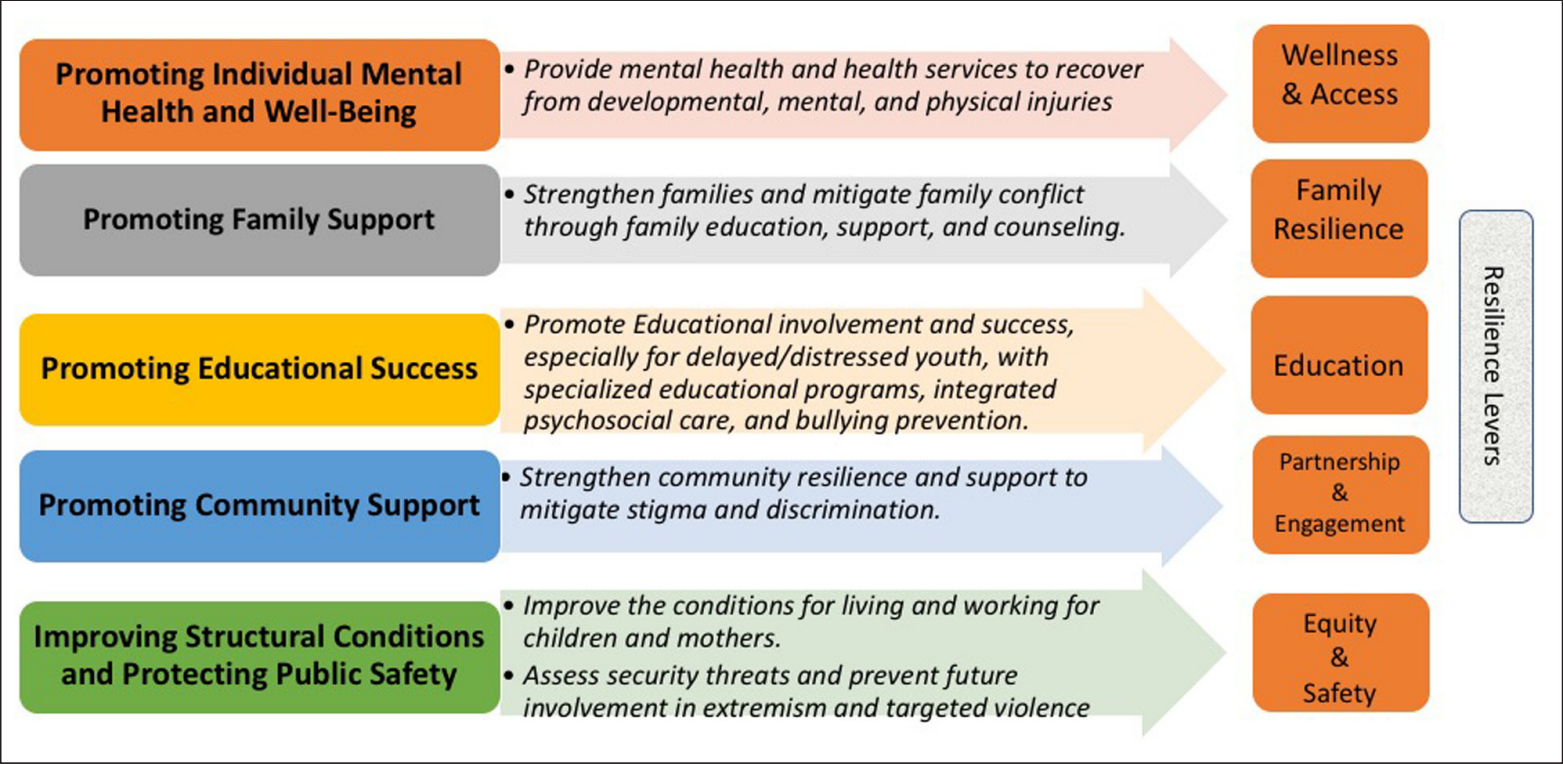
The levers of community resilience for the rehabilitation and reintegration of child returnees.

Finally, the RRIF also identifies how each of the goals corresponds to the known **levers of community resilience** [44] (Figure 4).

This rapid review also made it apparent that multiple components that are distinctive of child returnees from the IS are not addressed at all or adequately in the existing literature gathered. Specifically, the gaps in research knowledge that we identified concerned: 1) health and developmental problems in children due, for example, to illness, injury, or malnutrition [42,43,45]; 2) addressing family custody issues, where family members are in conflict about who the children should live with [45,46,47]; 3) addressing how parents, faith-based organizations, and possibly the state, should be educating children about faith and religion [48,49]; and 4) violence and radicalization risk assessment, prevention, and intervention [42,45,50,51]. As indicated above, for each of these topics, we found other literature to draw upon from areas that were outside the scope of this rapid review. Thus, in the development of RRIF, we also included some components that were not found in the existing literature on children exposed to trauma and adversity.

## Discussion and Recommendations

Rehabilitation and reintegration programs should be based on evidence of prior work with children exposed to trauma and adversity. RRIF defines a multi-level approach that encompasses promoting individual mental health and well-being, family support, educational success, community support, structural conditions and public safety.

The new framework can be compared to existing models of working with war-impacted communities, such as the IASC Guidelines or the WHO Service Organization Pyramid [52,53]. These existing frameworks each propose mixed levels of services, including self-care and primary care, instead of relying exclusively on specialists or psychiatric hospitals. Our pyramid applies this same organizing principle, while also incorporating several additional sectors necessary for the rehabilitation and reintegration of children, such as public safety, schools, and families. The new framework emphasizes a multilevel approach, implying that activities are needed at each level in order to succeed. As noted earlier, this framework may differ somewhat from the prior models, in that it does not necessarily imply the same proportionality (e.g. greater emphasis on lower levels of the model, which fits with the public health aspect of those other models). In that sense, it should be recalled that rehabilitation and reintegration is itself an act of tertiary prevention which is focused on a relatively smaller number of individuals (in the tens or hundreds in most countries).

Rehabilitation and reintegration programs should encompass building resilience to violent extremism through activities that enhance the levers of *wellness, access, family resilience, education, partnership, engagement, equity*, and *safety*. To facilitate buy-in and sustainability, this approach should also consider encompassing other threats, risks, and resources that the community identifies. In addition, exclusively focusing new services and supports on child returnees while neglecting other children’s needs could unintentionally create resentment and fuel stigma.

The framework developed through this rapid review can only be implemented through public-private partnership with intensive civil society involvement and multidisciplinary collaboration. Notably, the security-focused goal requires the leadership of security agencies but should also involve community policing approaches and the active, appropriate participation of civil society partners such as learning how to do violence risk assessments. This implies that security and civil society organizations and practitioners need to find additional ways to share information, cooperate, and collaborate. To successfully implement community-based programs that provide multilevel and multidimensional support to child returnees will require multiple actors from multiple sectors working collaboratively.

It is worth noting that most research included as the basis for this review was conducted in the U.S. or other high-income countries in the West. As such, issues of relevancy and adaptability will be important to consider when developing programming for returnees to low- and middle-income countries with diverse sociocultural contexts.

In many countries, especially low- and middle-income countries, efforts are needed to build the capacity of leadership and practitioners in government and civil society. Areas of need include locally focused training on: 1) trauma-informed mental health care; 2) developmental assessment and support; 3) specialized educational programs for children with educational difficulties or special needs; 4) violence extremism risk assessment and prevention; and 5) building community and family resilience.

Further research is needed to support rehabilitation and reintegration programs involving child returnees from the IS. That research should embody a rigorous longitudinal design to investigate the process and impact of rehabilitation and reintegration activities. It is especially important to build evidence in the gaps outlined above concerning health and developmental problems, family custody, faith and religion, and violent extremism assessment and prevention. To do so will require multidisciplinary research collaborations, combining clinical, community, security, and social sciences expertise.
